# A dedifferentiated solitary fibrous tumor of the parotid gland: a case report with Cytopathologic findings and review of the literature

**DOI:** 10.1186/s13000-019-0792-6

**Published:** 2019-02-18

**Authors:** Chien-Kuan Lee, Ken-Liao Liu, Sheng-Kai Huang

**Affiliations:** 1 Department of Pathology, Kung Tien General Hospital, Shalu, Taichung, Taiwan; 2 Department of Otorhinolaryngology, Kung Tien General Hospital, Shalu, Taichung, Taiwan

**Keywords:** Dedifferentiated solitary fibrous tumor, Parotid gland, FNA, Cytology, ERG

## Abstract

**Background:**

Solitary fibrous tumor (SFT) is a ubiquitous mesenchymal neoplasm but it rarely occurs in the parotid gland. The histological features are variable, with the majority having spindle cell morphology and non-specific branching (staghorn) ecstatic vascular pattern. SFT ranges from benign to overtly malignant. Dedifferentiation within SFTs represents an abrupt transition from a well-differentiated component to a high-grade area, the latter most often including poorly differentiated epithelioid/round cell or high-grade spindle cell morphology. To the best of our knowledge, dedifferentiated SFT in the parotid gland has not been previously reported.

**Case presentation:**

A 33-year-old woman presented with a soft tissue tumor in the right parotid gland that had been present for 6 months. Fine needle aspiration (FNA) cytology indicated epithelioid morphology in the dedifferentiated component of the tumor, along with metachromatic myxoid matrix. The tumor was initially interpreted as a salivary gland neoplasm of uncertain malignant potential (SUMP).Right partial parotidectomy was performed, and microscopic examination of the resected specimen revealed a malignant spindle cell tumor with a central epithelioid/anaplastic component. The tumor cells were diffusely positive for CD34, STAT-6 and FLI-1, and negative for pan-cytokeratin, CAM5.2, p63, S100 protein, CD31, SMA, and calponin.ERG and Ki67 immunostaining showed an accentuated nuclear staining pattern in the central dedifferentiated area. There was no overexpression of p53 or p16. The patient is currently undergoing regular follow-up and is 11 months postresection with no evidence of recurrence or distant metastasis.

**Conclusions:**

Unlike the typical spindle cell morphology of conventional SFTs, malignant SFTs can show areas of dedifferentiation mimicking an epithelial neoplasm. FNA of dedifferentiated SFTs of the parotid gland may show, a combination of atypical epithelioid cells and metachromatic myxoid/collagenous matrix, which is a less emphasized cytological feature of SFT and may lead to misdiagnosis as a more common parotid gland epithelial neoplasm.

## Background

Solitary fibrous tumor (SFT) is a mesenchymal neoplasm of presumed fibroblastic origin. Initially described in the pleura by Klemperer and Rabin in 1931 [1], it has also been frequently encountered in extrapleural sites virtually everywhere in the body. Although most SFTs pursue a benign clinical course, approximately 12–22% behave aggressively [2]. The 2013 WHO classification of soft tissue tumors defines malignant SFTs as hypercellular, mitotically active (≥4 mitoses per 10 high-power fields[HPFs]), with cytological atypia, tumor necrosis, and/or infiltrative margins. However, histological features do not reliably predict aggressive clinical behavior. As a result, risk stratification model using clinicopathologic features (tumor size, necrosis, mitotic activity and patient age)has been proposed and refined for better prediction of tumor metastasis [3]. Moreover, dedifferentiation, a phenomenon well-recognized in mesenchymal tumors such as well-differentiated liposarcoma, chondrosarcoma, chordoma, and osteosarcoma, has also been described in SFTs, posing a higher risk of tumor recurrence and/or metastasis [4]. The histologic features of the dedifferentiated component include epithlioid, round and/or spindle cells with increased mitotic activity necrosis and cystic degeneration [4].

Approximately 6% of SFTs occur in the head and neck region; occurrence in the parotid gland is rare, with only 31 cases reported in the English literature [5–12].While most of these cases showed benign histomorphology, 2 were histologically malignant. Here, we report an additional case of malignant SFT occurring in the parotid gland, with a discrete dedifferentiated/epithelioid component, unusual cytomorphologic features, and ERG expression by immunohistochemistry.

## Case presentation

**Fig. 1:**
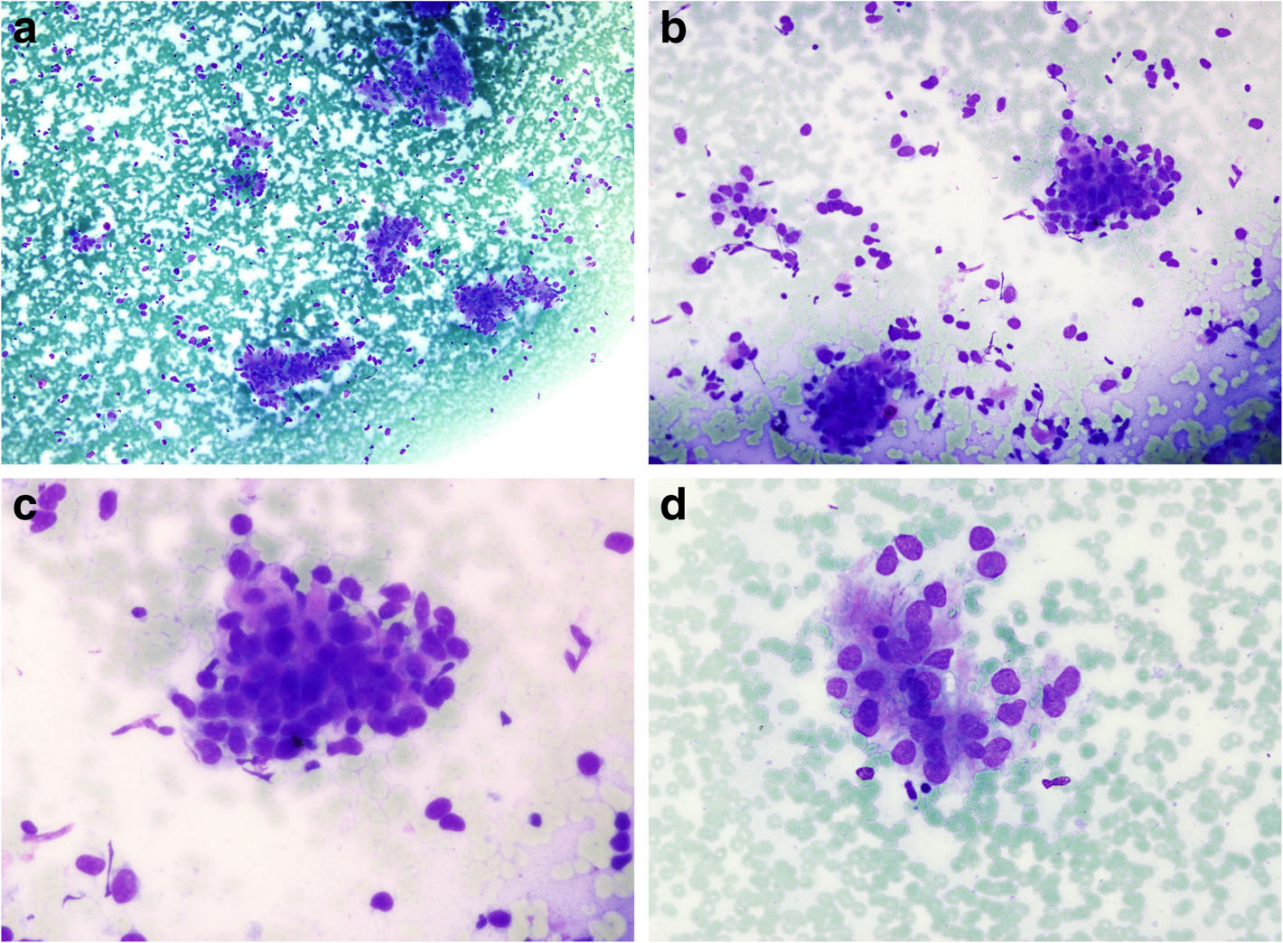
Cytomorphology of find needle aspiration. **a** and (**b**) Moderate cellularity with cellular clusters and single cells (Liu’s stain, 100x and 200x). **c** Epithelioid cells with eosinophilic cytoplasm and naked nuclei with nulcer atypia (Liu’s stain, 400x). **d** A loosely cohesive cluster with pink myxofibrillary matrix(Liu’s stain, 400x)

A 33-year-old woman presented with an elastic, non-tender mass over the right parotid area for 6 months. The patient had no significant past medical or surgical history. Physical examination showed a 3-cm round, palpable, immobile mass over the right parotid area. The overlying skin showed no sign of inflammation. There was no facial paralysis or cervical lymph node enlargement. Computed tomography of the head and neck with contrast revealed a 3.7 × 2.7 cm round mass over the right parotid gland with heterogeneous enhancement. There were some subcentimeter non-specific lymph nodes over bilateral level Ib and II. Preoperative fine needle aspiration (FNA) was performed and the smears were moderately hypercellular, with small to large cohesive tissue fragments, as well as scattered single cells in the background (Fig. 1a,b). Both the tumor clusters and single cells showed epithelioid morphology with an increased nuclear to cytoplasmic(N:C) ratio, round to oval nuclei, moderate nuclear pleomorphism and a lack of nucleoli (Fig. 1c). These atypical epithelioid cells were embedded in myxomatous and fibillary matrix with eosinophilic/light purple cytoplasm(Fig. 1d).No mitotic figures were found and there was no necrosis in the background. A diagnosis of salivary gland neoplasm of uncertain malignant potential (SUMP) was rendered. Right partial parotidectomy was then performed smoothly using an intraoperative neuromonitoring system without damage to facial nerve.

**Fig. 2:**
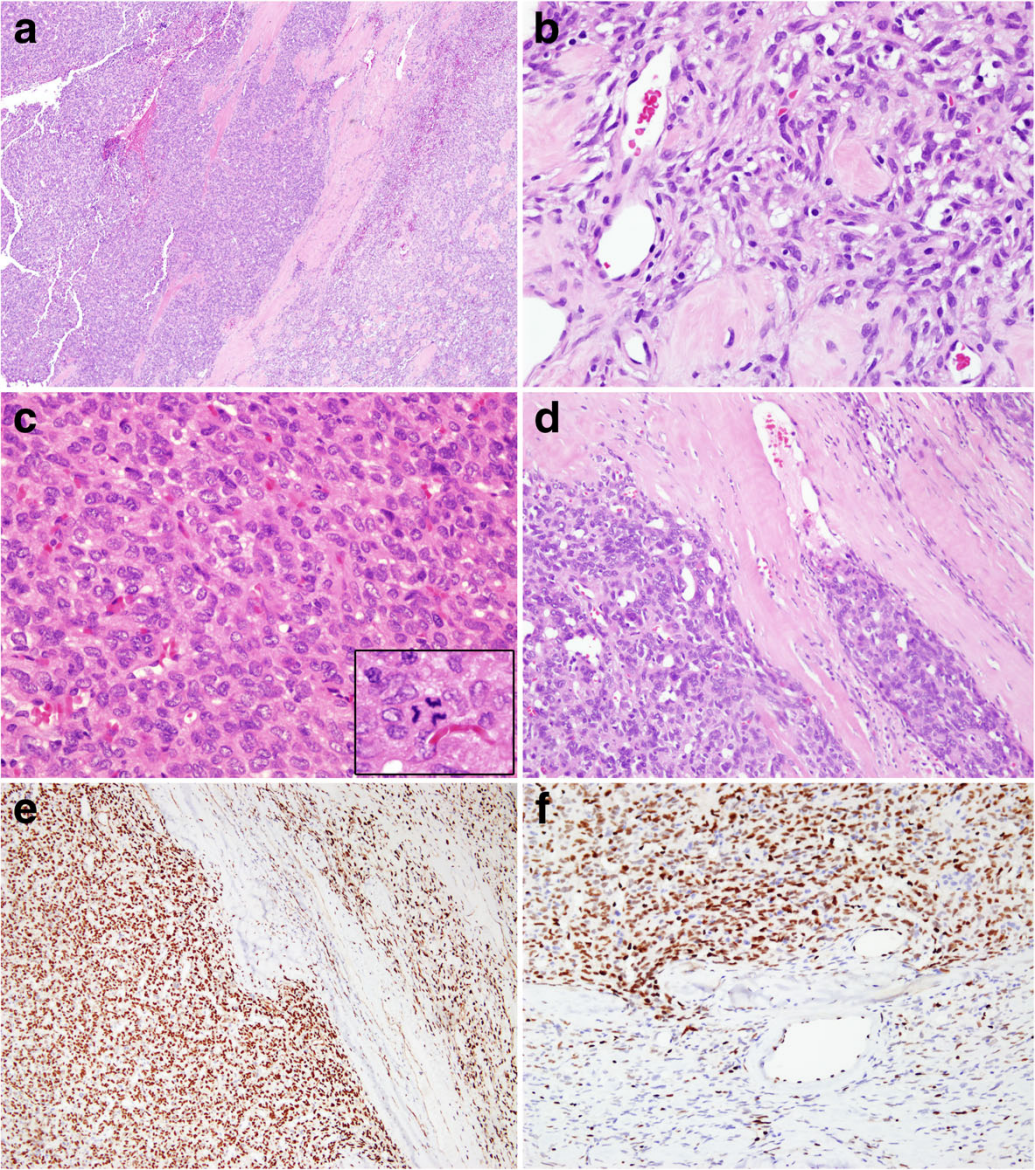
**a** Dedifferetiaetd component (left) separated by fibrous septa, with abrupt transition to conventional solitary fibrous tumor (right) (Haematoxylin and eosin stain, 100x). **b** Low-grade area showing spindle cell morphology with sclerotic stroma (Haematoxylin and eosin stain, 400x). **c** Dedifferentiated area exhibiting poorly differentiated epithelioid morphology. Inset shows atypical mitotic figure (Haematoxylin and eosin stain, 400x). **d** Angiolymphatic permeation of pleomorphic tumor cells within fibrous septa (Haematoxylin and eosin stain, 400x). **e** Positive immunohistochemical staining for STAT-6 in both dedifferentiated and convention SFT areas. **f** Strong nuclear staining of ERG in anaplastic epitheloid cells (upper half); attenuatedor absent expression in peripheral spindle cells (lower half)

Gross examination of the resected specimen revealed a well-circumscribed, unencapsulated, gray-brown soft tissue mass, measuring 3.5 × 2.1 × 2.0 cm. On cross-section, the tumor was homogenous and tan-brown with occasional small hemorrhagic cysts. In addition, a nodular expansion with indistinct fibrous capsule was found within the tumor, reminiscent of the “nodule-in-nodule” appearance of hepatocellular carcinoma. Microscopically, the tumor had a peripheral low-grade area and a central high-grade area. The low-grade area was predominantly composed of spindle cells with varying cellularity and focal reticular pattern, interspersed with sclerotic stroma, rounded vessels and infiltration of the adjacent tissue(Fig. 2b). The tumor cells had bland, round to short spindle cell morphology, with minimal cytoplasm and vesicular chromatin. The central hypercellular area was sharply demarcated by thin fibrous septa and comprised enlarged epithelioid tumor cells (Fig. 2c) with moderate nuclear atypia arranged in a sheet-like pattern with hemangiopericytoma(HPC)-like hyalinizing vessels. Nuclear pleomorphism with hyperchromasia, a high N:C ratio, clumped chromatin, irregular nuclear membrane, and inconspicuous small nucleoli were observed in the high-grade component. There was an abrupt transition between these two components (Fig. 2a). The mitotic rate was up to 5 per10 HPFs, and atypical mitosis was observed (Fig. 2c). Angiolymphatic permeation (Fig. 2d)and infiltrative growth were also present. The resection margins of the tumor involved both the low- and high-grade areas.

Immunohistochemically, the tumor cells were diffusely positive for CD34, STAT-6 and FLI-1, but negative for pan-cytokeratin, CAM5.2, p63, S100 protein, CD31, SMA, and calponin. ERG and Ki67 immunostaining showed an accentuated nuclear staining pattern in the central dedifferentiated area. The Ki-67 labeling index was around 20% in the hypercellular area, and 3% in the loose short spindle cell area. There was no overexpression of p53 or p16.This patient received adjuvant radiation therapy with 70Gy to the right parotid area due to a microscopically positive resection margin (R1) and has been followed-up for 11 months, with no signs of recurrence or distant metastasis.

## Discussion and conclusions

SFT is a soft tissue tumor that appears only rarely in the parotid gland, with 31 previously reported cases [5–12]. Age of occurrence ranges from 11 to 79 years, and it has no gender predilection. Clinically, these tumors usually present as painless, firm, well-defined, slow-growing masses a few months to a few years in duration [5]. Obstructive sleep apnea is not uncommon and may be related to tumor compression [8]. On computed tomography, SFTs can be hyperintense with respect to adjacent tissues, with heterogenous-enhancement after contrast administration. Magnetic resonance imaging usually shows a signal similar to any soft tissue tumor, with intermediate signal intensity on T1-weighted images and enhancement on T2-weighted images [9].

Previously describd SFTs of the parotid gland were macroscopically well circumscribed, with the exception of one malignant case. The tumor were partially to fully encapsulated with a firm, gray-white cut surface, and ranged from 1 to 18 cm (average 4.8 cm, median 4 cm). Histologically, SFT has a wide range of features, from cellular neoplasms to predominantly fibrous lesions, and intermediate forms between the two ends of the spectrum. Fibrous forms of SFT are characterized by alternating hypercellular and hypocellular fibrous areas. Cellular forms of SFT resemble what have traditionally been called Hemangiopericytoma (HPC). Most parotid SFTs previously described in literature were of those “fibrous variant”, with one case categorized as adipocytic variant [5, 10].

Like SFTs occurring in other anatomic sites, parotid SFTs may also show malignant features infrequently; the two malignant cases described in the literature exhibited increased cellularity, marked atypia, high mitotic activity, but no area of necrosis [13, 14]. Our case is the third case of malignant parotid SFT reported to date. Interestingly, our case was characterized by a central dedifferentiated area in addition to malignant histology. Dedifferentiation is a well described phenomenon in soft tissue and bone tumors, but its occurrence within SFT has just recently been described by Mosquera et al. [4]. Dedifferentiation either arises de novo or develops in a recurrece of a previous well-differentiated tumor. Similar to other dedifferentiated sarcomas, abrupt transition between low-grade and high-grade/anaplastic area is typically observed in dedifferentiated SFT. The latter component is morphologically nondistinctive, most often showing epithelioid, round cell or spindled morphology with increased mitotic activity, necrosis, and cystic degeneration [4]. Rarely, “transdifferentiation”to non-mesenchymal lineage, such as neuroendocrine carcinoma and squamous differentiation, has been reported [15].

SFT has been a diagnostic challenge for cytopathologists, not only because of the morphological overlap with various soft tissue tumors, but also because of the variability in the growth pattern [16, 17].Depending on which area of the tumor is sampled, the cellularity of the FNA can range from scanty to quite cellular, with a broad spectrum of cell types, ranging from spindle/fibroblastic to epithelioid/round cells [16, 17]. The spindle /bipolar cells have elongated nuclei and slender cytoplasmic processes, which are found predominantly in benign and sclerotic areas. The dendritic/stellate cells have oval nuclei and thick cytoplasm with dendritic processes and are more common in smears with abundant cellularity. The presence of abundant vessels in smears may be helpful in the cytologic diagnosis of SFT [17]. Findings of nuclear pleomorphism, necrosis, or mitotic figures may indicate malignancy; however, it is extremely difficult to distinguishmalignant SFTs from conventional SFTs by FNA. As the former may have zones that are morphologically identical to those ofthe latter, aspiration sampling may be misleading [18, 19]. Ali et al. proposed using a predominance of single cells as a feature that favored malignancy [20], whereas Bishop et al. found a general lack of single cells in their series [18]. A combination of cohesive tumor clusters and single cells was observed in our smears (Fig. 1a,b). Of note, loosely cohesive spindle cell tissue fragments associated with abundant metachromatic myxoid/collagenous matrix, is a less emphasized but prominent cytological finding in both benign and malignant SFTs [16, 18].

Although there is limited information in the literature regarding the cytology of parotid SFTs, most reported cases are histologically fibrous and show spindle cell cytomorphology, suggesting a spindle cell neoplasm [5, 13, 17]. However, the combination of myxofibrillarystroma and atypical basaloid cell morphology (as seen in our case, Fig. 1c,d)has not been described in the literature, which could be a potential diagnostic pitfall due to the morphological overlap with epithelial neoplasms of the parotid gland on FNA. A broad differential diagnosis of cellular basaloid neoplasm should be considered, including cellular pleomorphic adenoma, carcinoma ex pleomorphic adenoma, epithelial-myoepithelial carcinoma, and basal cell adenoma/adenocarcinoma. As in our case, the clinical finding of a parotid gland neoplasm, absence of spindle cells, and the sampling of dedifferentiated/ epithelioid area, lead to the diagnosis of SUMP. Nevertheless, in general practice, as long as a diagnosis of “neoplasm” is used, appropriate surgical management will be implemented regardless of whether a diagnosis of primary salivary gland epithelial neoplasm or mesenchymal neoplasm is rendered [5]. Moreover, the cell block could be key to identifying the HPC-like pattern and can be a source for the application of immunohistochemical stains [19, 20].

The differential diagnosis of SFT involving parotid gland includes a variety of spindle cell neoplasm, and many display similar histology. For example, deep benign fibrous histiocytoma may closely resemble SFT; both are well-circumscribed, contain HPC-like vessels and may express CD34. However, deep benign fibrous histiocytoma usually shows a storiform growth pattern rather than the patternless architecture and alternating hypocellular and hypercellular areas of SFT. Spindle cell lipoma occurs predominantly in the neck, upper back and shoulder and is usually strongly and diffusely positive for CD34. Although ropy collagen bundles, short stubby spindle cells and a variable adipocytic component are characteristic of spindle cell lipoma, cellular variants of spindle cell lipoma lacking adipocytes are especially difficult to distinguish from SFT. In contrast to SFT, cellular schwannoma and spindle cell melanoma are both diffusely positive for S100 protein. Traditionally, immunohistochemical markers such as CD34, CD99, and BCL2 are examined, but none is sufficiently sensitive or specific to distinguish these tumor types. This has been greatly simplified by the immunohistochemical detection of STAT6, a very sensitive and specific marker for SFT, which identifies the NAB2-STAT6 fusion product. Strong nuclear expression of STAT6 is seen in more than 95% of SFT, whereas low-level cytoplasmic and nuclear expression is typically seen in other mesenchymal tumors [21].Of note, amplification of STAT6 at 12q13 and STAT6 protein expression is detected in a subset of dedifferentiated liposarcomas (11%, 4/35 cases) [22], and may be a potential pitfall in the differential diagnosis. In addition, the central dedifferentiated area in our case mimicked a malignant epithelial neoplasm, such as carcinoma ex pleomorphic adenoma with prominent stroma. Lack of pan-cytokeratin and absence of myeoepithial markers (e.g. S100 protein, calponin) can help exclude the differential diagnosis.

In the case of dedifferentiated SFT, the abrupt transition to a morphologically anaplastic component is often accompanied by loss of CD34 expression and strong expression of p53 and p16 [4]. In addition, patchy or negative STAT6 expression is frequently seen in the high-grade regions. Therefore, although STAT6 immunohistochemisty correctly identifies all conventional and malignant SFTs, it is not always detected in the dedifferentiated component [23]. RT-PCR may be helpful in such cases since the NAB2/STAT6 fusion is retained in all STAT6-negative dedifferentiated tumors [23, 24]. The immunoprofile in our case showed retained expression of CD34 and STAT6 and there was no overexpression of p53 or p16 in the dedifferentiated component.

Unexpectedly, in this case, the majority of epithelioid cells in the dedifferentiated area exhibited a strong nuclear staining of ERG, a lineage specific marker of endothelial differentiation [25], whereas its expression was attenuated in the peripheral spindle tumor cells (Fig. 2f). The combination of ERG and other vascular markers (i.e. CD34, FLI-1) could be a diagnostic pitfall, suggesting a vascular neoplasm; however, the diagnosis of SFT was supported by the typical SFT morphology at the periphery, the strong expression of STAT6 and the absence of vasoformative morphology and CD31. The significance of ERG expression in the dedifferentiated component is neither clear nor equivalent to heterologous endothelial phenotypes, and requires study of more cases to address whether this is simply an aberrant expression pattern.

As mentioned above, the diagnosis of SFT has been simplified by the the recent discovery of recurrent NAB2-STAT6 gene fusion on chromosome 12 and the subsequent upregulation and overexpression of STAT6, which can be detected by immunohistochemistry [21]. There are several variants of the fusion with a range of breakpoints; NAB2ex4-STAT6ex2/3, appears to be the most predominant fusion variant, causing a classic fibrous SFT phenotype with an intrathoracic location in elderly. In contrast, the 2nd most common variant, NAB2ex6-STAT6ex16/17, tends to be detected in deep-seated and extrathoracic sites, affecting younger patients [26]. No recurrent or specific fusion type associated with dedifferentiated SFT has been identified [27].

Identifying which tumors will behave aggressively is problematic in conventional SFT. There is no strict correlation between the morphobiological characteristics of a tumor and its clinical course, as histologically benign tumor can occasionally recur and metastasize after a long time [3].Malignant SFTs are usually hypercellular lesions with increased mitoses (≥4 per 10 HPFs), variable cytological atypia, tumor necrosis, and/or infiltrative margins, with mitotic activity being considered the most reliable criterion in microscopic examination. However, Demicco et al. showed that mitotic activity alone might not be enough to accurately discriminate aggressive tumors, and is just one factor in the assessment. Although mitotic activity has been shown to be an independent risk factor, it might overestimate the risk of metastasis by risk stratification analysis. In their recent study, a refined four-tier risk stratification model for SFT was proposed, incorporating patient age, tumor size, mitotic activity, and tumor necrosis to predict risk of metastasis [3].Our case was stratified into the low-risk class, suggesting a very low likelihood of metastasis. Of note, this risk score is specifically designed to assess metastatic risk and not prediction of recurrence or overall survival. While initial efforts using *NAB2-STAT6* fusion variants as molecular predictors show no correlation between fusion type and disease-free survival [26, 28], TERT promoter mutations, reported to be present in 20–30% of SFTs,have emerged as another potential driver of aggressive behavior. Some authors suggest that TERT promoter mutation status might not be a reliable predictor of clinical outcome by itself; however, by integrating it into existing multivariable risk stratification, it might help refine outcome predictions in intermediate-risk cases [29, 30].As for dedifferentiated SFTs, there is limited available data regarding their clinical behavior and molecular findings, but it seems that these lesions are likely to behave more aggressively than conventional malignant SFTs [4, 27].

Complete excision with negative surgical margins is the first choice of treatment and leads to a good prognosis [5]. Preoperative embolization can also be employed in highly vascular tumors [6]. Postoperative radiation and/or chemotherapy in cases of incomplete resection or with malignant histological features may be considered [6, 7]. Most reported cases in the parotid gland showed no evidence of disease at follow-up [5]; however, two patients had local recurrences or aggressive behavior despite benign histology or negative resection margins [11, 13]. Only two histologically malignant cases of SFT of the parotid gland have been reported in the literature; one had pulmonary metastasis at the time of diagnosis [31], whereas the other patient survived for 6 years free of disease [14]. Long term follow-up with clinical and imaging examinations (ultrasonography and/or computed tomography) for at least 3 to 5 years is recommended [12].

We herein reported the cytopathological findings of dedifferentiated SFT of the parotid gland. Unlike the typical spindle cell morphology in conventional SFTs, malignant SFTs can show areas of dedifferentiation mimicking an epithelial neoplasm. On FNA of dedifferentiated SFT of the parotid gland, the combination of atypical epithelioid cells and metachromatic myxoid/collagenous matrix, a less emphasized cytological feature of SFT, may lead to misdiagnosis as a more common parotid gland epithelial neoplasm.

## Abbreviations

(DFSP): Dermatofibrosarcoma protuberans
(DLPS): Dedifferentiated liposarcoma
(FNA): Fine needle aspiration
(GIST): Gastrointestinal stromal tumor
(HPC): Hemangiopericytoma
(IONM): Intraoperative neuromonitoring system
(LGFMS): Low grade fibromyxoid sarcoma
(MPNST): Malignant peripheral nerve sheath tumor
(SFT): Solitary fibrous tumor
(SUMP): Salivary gland neoplasm of uncertain malignant potential
